# Analysis of the Impact of Sustained Load and Temperature on the Performance of the Electromechanical Impedance Technique through Multilevel Machine Learning and FBG Sensors

**DOI:** 10.3390/s21175755

**Published:** 2021-08-26

**Authors:** Ricardo Perera, Lluis Torres, Francisco J. Díaz, Cristina Barris, Marta Baena

**Affiliations:** 1 Department of Mechanical Engineering, Technical University of Madrid, 28006 Madrid, Spain; franciscojavier.diaz.nunez@alumnos.upm.es; 2 Analysis and Advanced Materials for Structural Design (AMADE), Polytechnic School, University of Girona, 17003 Girona, Spain; lluis.torres@udg.edu (L.T.); cristina.barris@udg.edu (C.B.); marta.baena@udg.edu (M.B.)

**Keywords:** structural health monitoring, hierarchical clustering, k-means clustering, PZT sensors, FBG sensors, sustained load, temperature, NSM-FRP strengthening

## Abstract

The electro-mechanical impedance (EMI) technique has been applied successfully to detect minor damage in engineering structures including reinforced concrete (RC). However, in the presence of temperature variations, it can cause false alarms in structural health monitoring (SHM) applications. This paper has developed an innovative approach that integrates the EMI methodology with multilevel hierarchical machine learning techniques and the use of fiber Bragg grating (FBG) temperature and strain sensors to evaluate the mechanical performance of RC beams strengthened with near surface mounted (NSM)-fiber reinforced polymer (FRP) under sustained load and varied temperatures. This problem is a real challenge since the bond behavior at the concrete–FRP interface plays a key role in the performance of this type of structure, and additionally, its failure occurs in a brittle and sudden way. The method was validated in a specimen tested over a period of 1.5 years under different conditions of sustained load and temperature. The analysis of the experimental results in an especially complex problem with the proposed approach demonstrated its effectiveness as an SHM method in a combined EMI–FBG framework.

## 1. Introduction

Effective strengthening techniques, such as externally bonded reinforcement (EBR) or near-surface mounted (NSM) using FRP materials, have been used in the last decade to increase the load carrying capacity of reinforced concrete (RC) structures. The NSM technique yields better bond performance and other potential advantages such as a better protection of FRP against external actions and minimal changes in the aesthetics of the structural elements [1,2,3].

The flexural and shear behaviors of RC beams strengthened with NSM-FRP under static loading conditions have been widely studied in the literature. However, although several studies have been performed regarding the combined effects of sustained load and temperature on the bond response of NSM-CFRP laminates in concrete elements [4,5,6,7], studies devoted to the global response of the strengthened structure under those same conditions acting simultaneously are practically nonexistent. This paper presents an experimental study to examine the long-term performance of a RC beam strengthened with NSM-FRP and subjected to a sustained load in conjunction with changing temperatures. 

Furthermore, considering the uncertainty regarding the failure mode of this type of structure and the high probability of the occurrence of brittle and sudden failure, a structural health monitoring (SHM) methodology should also be implemented to predict any deterioration in the structure at its earliest stages and its evolution. 

Continuous measurement of strain represents an important part of SHM systems. Measured strains provide valuable information about the integrity of a structure. Fiber Bragg grating (FBG) optical sensors, unlike conventional electric strain gauges, are robust enough to sustain harsh working conditions and are immune to noise. Although damage modifies the load carrying capacity of a structure and creates modified strains, the strain variations due to the incipient damage are difficult to determine. The use of a lead zirconate titanate (PZT)-based electromechanical impedance (EMI) technique is more suitable and reliable for identifying incipient damage [8,9]. However, although structural damage changes the impedance signature, other factors such as variations in temperature can also cause the signatures to change as well [10,11]. This can lead to a false positive diagnosis. To avoid this, temperature compensation techniques based on the use of a pattern of impedance measurements performed on the structure at various thermal levels are applied [12,13,14,15]. FBGs are intrinsically sensitive not only to the strain but also to temperature; thus, the combination of FBG and PZT technologies in combination with suitable data processing techniques might provide a suitable methodology to identify incipient damage in the presence of temperature variations and deserve to be explored.

Machine learning (ML) approaches are an interesting alternative way to detect hidden patterns in monitored data, and therefore, to separate the baseline from any anomalies experienced by the structure, either due to mechanical deterioration or change in temperature. This kind of technique might identify complex patterns related to different stages of the tested structure. The most common supervised machine learning algorithms are neural networks [16,17], support vector machines [18,19,20], and random forest [21,22], among others. In supervised machine learning algorithms, the possible responses of the problem are known.

In contrast, in unsupervised ML algorithms, the response of the problem is unknown. Clustering techniques are widely used in many practical applications for dimensional reduction [23,24,25] and are included within this group. The objective of a clustering analysis is to partition a data set into clusters such that the data points within the same cluster are similar, while data points in diverse clusters are different from each other. By using clustering analyses, the intrinsic structure of a data set can be captured.

This paper proposes a damage detection approach for NSM-FRP strengthened RC specimens subjected to sustained loading and variable temperature, based on the combined use of the PZT-EMI technique, FBG sensors and data processing based on multilevel clustering analysis. A concrete specimen strengthened with the NSM-FRP technique was subjected to different levels of sustained load for various time periods and at different temperatures during a test campaign of almost two years. The strain and temperature were continuously measured using FBG sensors. In the same way, after each sustained loading period or important temperature variation, an EMI test was performed with different PZT sensors located at different areas of the beam. FBG sensors and electromechanical impedance in conjunction with multilevel ML techniques have been employed independently in the past. We think that the combined use of FBG and PZT sensors in conjunction with clustering ML results in operational benefits for the monitoring of a problem in which the effects due to mechanical damage and temperature variations are coupled.

## 2. Clustering

Clustering analysis captures the intrinsic nature of a data set by making it possible to classify and categorize data in different groups according to their similarity. Within the different categories of clustering algorithms, hierarchical and k-means are the two most used types of classification [26,27]. K-means is used when the number of classes is fixed, while hierarchical clustering is used for an unknown number of classes. 

### 2.1. Hierarchical Clustering

Hierarchical clustering is one of the most commonly used methods for clustering data. By using this algorithm, the data set is divided into different clusters by iteratively merging or splitting clusters based on a dendrogram. Clustering algorithms provide information about the data to be classified in a compact and graphical way. They can be agglomerative or divisive depending on the procedure used to create the dendrogram. Agglomerative clustering follows a bottom-up strategy, in which, each data point is initially assumed to be a cluster and then it iteratively merges the two most similar clusters in terms of an objective function until the final dendrogram is obtained. By contrast, divisive clustering follows the opposite approach. All data points are considered initially as one cluster and then, iteratively, the selected cluster is partitioned into two new subclusters.

An agglomerative methodology is computationally more advantageous and more frequently applied. As this approach involves merging the two most similar clusters at each step, the choice of a suitable similarity measure or distance function is important for the final result. Different alternatives are possible, such as single linkage (SL), complete linkage (CL) and all-pairs linkage (APL) clustering.

As SL and CL are sensitive to noise and outliers, APL has been used in this study because of its higher robustness.

### 2.2. K-Means Clustering

K-means clustering is an unsupervised machine learning algorithm that groups clusters, sample points, or observations with a certain likeness to reveal hidden patterns. In this algorithm, the number of clusters must be defined a priori by the user. Then, an initial pattern of K clusters is randomly selected and each sample point is allocated to one of the produced clusters, using its minimum Euclidean distance from the center of that cluster as a reference. This process is repeated iteratively by updating the centroid of each cluster and the observations allocated in it, until the error is minimized and a suitable number of clusters is obtained. 

In this work, to apply k-means clustering, the 3rd order moments of the real part of the measured impedance responses have been chosen as the characteristic feature to process the data set.

(1) m3=∑i=1nRe(Z(ωi))×ωi3×dωi

where Re(Z(ωi)) is the real part of the impedance at the ith frequency point and *n* is the number of frequency points in the impedance spectrum.

### 2.3. EMI-Clustering Combined Approach

In the EMI technique, PZT transducers bonded to the structure using high strength epoxy are electrically excited with an impedance analyzer in a user-defined frequency range for measuring the electromechanical admittance (inverse of the impedance). This admittance (Y), comprising a real part termed conductance (G) and an imaginary part termed susceptance (B), is captured in the form of signatures with respect to the exciting frequency operating in sweep mode and is directly related to the mechanical impedance of the structure [28]. This means that the G and B signatures vary with any alteration in the mechanical characteristics of the host structure, and therefore, are useful for assessing its condition. Furthermore, PZT transducers are pyroelectric, which means that temperature variations will also affect their electrical impedance.

If we place PZT sensors in different zones of the specimen to be analyzed, the impedance measurements captured from each sensor at different stages will serve to identify the anomalies experienced by the specimen near the sensors. These anomalies can be provided by the progressive deterioration of the structure but also by the environmental variability and the identification of each of these would be desirable. 

The application of a clustering strategy, either using hierarchical or k-means or both jointly, might be extremely useful for solving this problem because of its capacity to abstract valuable statistics from experimental data archives based on impedance in an unsupervised training framework. Clustering algorithms might partition data points according to the defined similarity, and therefore, would be useful for the classification of the data across all loading stages.

## 3. Experimental Program

The experimental program consisted of the study of the behavior of a RC beam strengthened with an NSM-FRP sheet that was subjected to different levels of sustained load in conjunction with different temperature levels. The study was conducted over a period of more than 1.5 years. The following subsections outline the details of the beam, the experimental protocol, as well as the instrumentation used during the different tests. 

### 3.1. Experimental Beam and Sustained Load

**Figure 1:**
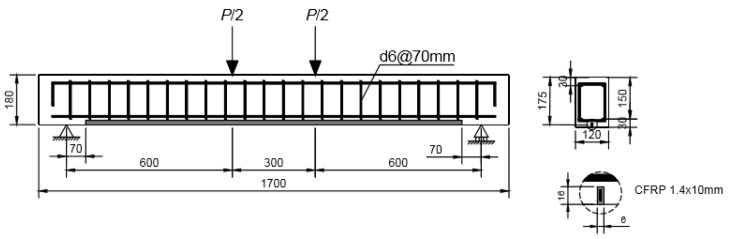
Experimental beam—geometry and reinforcement.

One 120 mm × 150 mm RC beam was used to perform the experimental program. The geometric dimensions and the reinforcement layout in the section are illustrated in Figure 1. The material properties of the concrete, steel reinforcement, and unidirectional FRP composite are as follows: (a) for the concrete, the elastic moduli, compressive strength and tensile strength were taken as E=26 GPa, $f_{c}=30\;\mathrm{MPa}$, and $f_{ct}=3\;\mathrm{MPa}$, respectively; (b) for the steel reinforcement, the elastic moduli and elastic limit were taken to be E=210 GPa, and $f_{y}=50\;\mathrm{MPa}$, respectively; (c) for the FRP reinforcement, the elastic moduli and the ultimate strength were taken to be E=170 GPa, and $f_{fu}=2550\;\mathrm{MPa}$, respectively; the width and the height of the FRP laminate were 1.4 mm and 10 mm, respectively.

**Figure 2:**
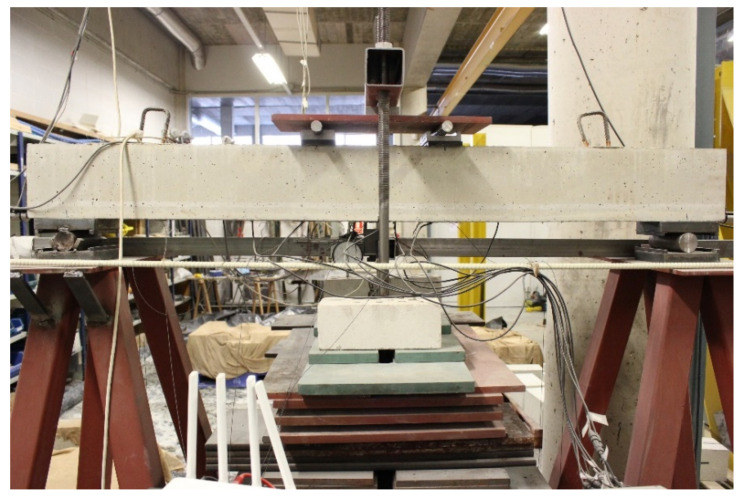
Configuration of the experimental test.

The beam was subjected to sustained load in four-point bending (Figure 1) and to temperature variations. A custom-made loading frame was utilized to apply the sustained load to the beam using perforated steel hollow sections (Figure 2). Four levels of sustained load were designed to represent the cracking load plus 15%, 35% and 100%, and the start of the steel reinforcement yielding of the strengthened beam, respectively. A fifth level of load was applied for which any increment in internal tensile force was practically and uniquely supported by the FRP reinforcement. The beam was exposed to room temperature during the sustained load tests. The increments in temperature on the beam were provided by heaters distributed uniformly along the length of the beam and were applied while the beam was not subjected to any sustained load. 

### 3.2. Test Setup and Instrumentation

The applied load was controlled by the weight of the materials put on the custom-made loading frame. The midspan deflection was recorded by a linear variable differential transformer (LVDT) and an electrical resistance strain gauge was installed to measure the compression strain at the midspan.

**Figure 3:**
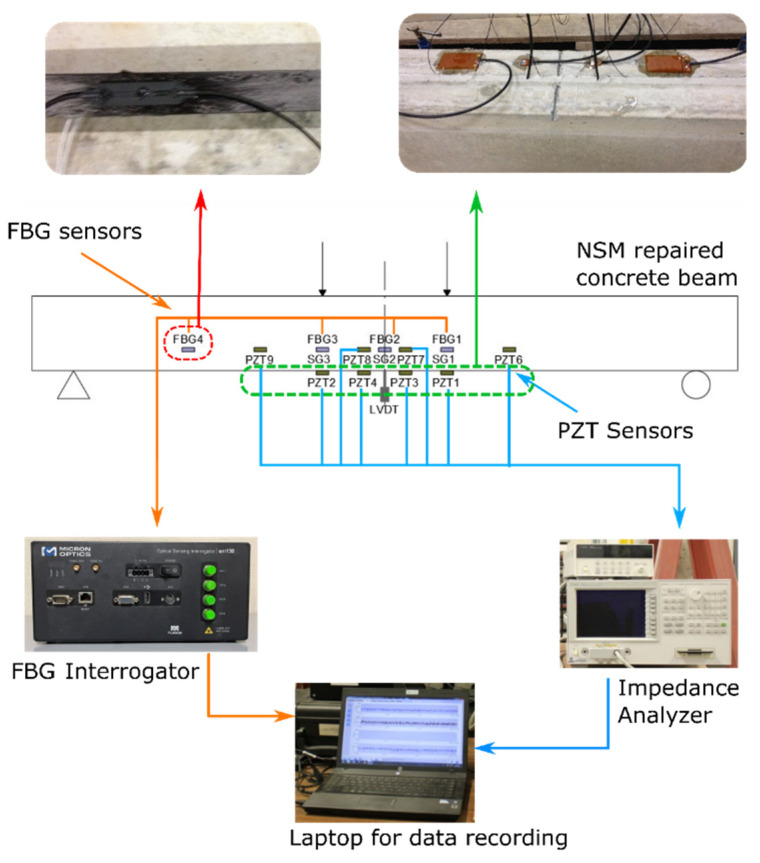
Experimental setup.

The performance along the FRP sheet was monitored by three FBG sensors, FBG1, FBG2, and FBG3 glued to the FRP laminate (Figure 3). The sensors were os3200 FBG sensors from Micron Optics. The FBG sensor working principle was modelled by the following relation [29]:

(2) $$\lambda_{B}=2n_{\mathrm{eff}}\Lambda$$

where λ_B_ is the Bragg wavelength, n_eff_ is the effective refractive index, and Λ is the Bragg grating period. The FBG sensor consists of a periodic modulation of the refractive index along a certain length of the optical fiber, which is known as grating. When a FBG sensor is subjected to an axial strain or temperature change, the peak wavelength shifts proportionally to the variations in these two parameters, which means that the FBG sensor can be utilized as a temperature or strain-sensing unit. 

Considering that some temperature variations will occur in our experiment, an os4100 FBG sensor from Micron Optics (Atlanta, GA, USA), FBG4 sensor in Figure 3, specifically designed to provide temperature compensation data for strain measurements from FBG-based strain gauges, was installed on the same structure. In this way, the effects due to the temperature were filtered for FBG1, FBG2, and FBG3.

The main goal of this work was to study the performance of the NSM-FRP RC beam under different conditions of sustained load and temperature. Hence, the change in the impedance across the different stages of the beam was also used. Impedances were captured from PZT patches bonded to the concrete tensile surface and to the FRP sheet; 61 mm × 35 mm × 0.5 mm and 16 mm × 13 mm × 0.5 mm PZT patches were used [30]. The terminals of the PZTs were attached to an impedance analyzer (Agilent HP 4192A (Agilent Technologies, Madrid, Spain)) for acquiring the EMI signatures, such as that shown in Figure 3. The EMI signatures were acquired in the range of 10–100 kHz, with a step interval of 12.5 kHz. 

According to Figure 3, PZT1 and PZT2 were glued to the concrete surface and correspond to type P-876.A12 DuraAct Patch sensors, while PZT3 to PZT9 correspond to P-876-SP1. PZT3 and PZT4 were bonded to the concrete surface while PZT6 to PZT9 were bonded directly to the FRP sheet. The PZT patches were manufactured to grade PIC151 and their properties are listed in Table 1.

An epoxy adhesive was used to bond PZT and FBG sensors to the specimen.

### 3.3. Experimental Protocol

To investigate the influence of the mechanism of the action of sustained load and temperature variations on the damage characteristics of the strengthened RC beam, the beam was experimentally tested for more than 1.5 years under different conditions of load and temperature, as shown in Table 2.

In general, except for the last loading stages, the methodology was as follows: (a) execution of one or several sustained load tests under the same predetermined load level and considering different durations; (b) unloading the specimen; (c) heating the specimen and maintenance of that temperature for 1–3 days; (d) removal of the heaters so that the beam reached room temperature. The beam stayed under room temperature and without loading for 1–3 days; (e) after step (d), the beam was subjected to a new sustained load test and the abovementioned procedure was repeated.

The values of the applied sustained vertical load were 8, 9.3, 13.7, 17.7, and 19.6 kN. As mentioned previously, the first three levels correspond to the cracking load plus 15%, 35%, and 100%, respectively, the fourth level is associated with the steel reinforcement yielding, and finally, at the fifth level, because of the high level of yielding reached in the steel reinforcement, any new increment in internal tensile force was practically supported by the FRP reinforcement.

### 3.4. Comparison between Experimental Tests

The differences between impedance spectra provide a visual qualitative assessment of the variations between tests. Different damage metrics can be used to compare the quantitative variations between different frequency spectra based on the impedance. These damage metrics are statistical measures that allow us to identify deviations between two compared data sets associated with two states of the structure. Commonly used damage metrics include the root mean square deviation (RMSD), the mean absolute percentage deviation (MAPD), and the correlation coefficient deviation (CCD). In this work, the RMSD coefficient was used and it was computed between two consecutive stages as [11]:

(3) RMSD(%)=∑i=1N[Re(Z1(ωi))−Re(Z0(ωi))]2∑i=1NRe(Z0(ωi))2·100

where $Z_{0}\left(\omega_{i}\right)$ is the impedance spectra of the PZT measured in a previous stage, $Z_{1}\left(\omega_{i}\right)$ is the spectra corresponding to a subsequent stage, and N is the number of frequency data points in the EMI spectra.

In the same way, to better visualize the results obtained from k-means clustering, a damage indicator is defined. If k-means clustering is performed previously at each test, it is possible to define a damage indicator, D, associated with each ith test or observation by computing the Euclidean distance to the centroid k of each cluster defined for the test:

(4) $$D_{k}\left(s_{i}\right)=\left(s_{i}-\mu_{k}\right)\sum_{k}^{-1}\left(s_{i}-\mu_{k}\right)$$

where s_i_ is the observation vector associated to the ith test and µ_k_ and Σ_k_ are the mean vector and the covariance matrix, respectively, computed for each cluster k; the s-vector includes the damage indicator for each sensor.

Once D is obtained for each cluster, the damage indicator for each ith observation is defined as the smallest value computed for all clusters:

(5) $$D\left(s_{i}\right)=\min\left[D_{k}\left(s_{i}\right)\right]$$

By using Equation (5), a damage index is obtained for each sensor and test. 

In Equations (4) and (5), the clusters taken as the baseline to compute µ_k_ and Σ_k_ can be those defined from the initial healthy state or any other that might be appropriate. 

## 4. Health Monitoring Using FBG and PZT Sensors 

### 4.1. Temperature/Strain Measurement Using FBG Sensors

**Figure 4:**
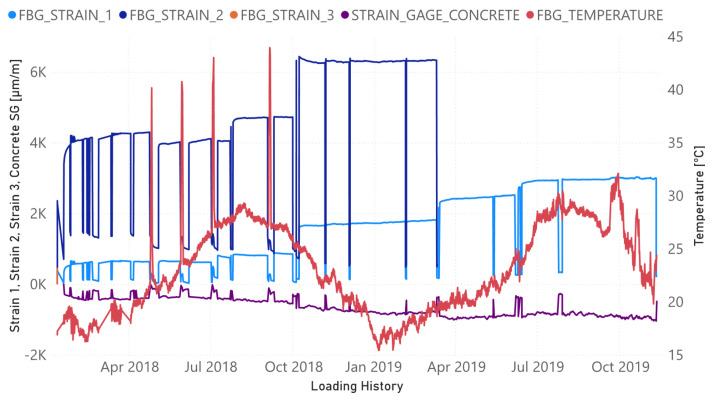
Evolution of tensile and compressive strains and temperature along the loading history.

Readings from the FBGs, such as those shown in Figure 3, were taken continuously. Figure 4 shows the temperature/strain evolution of FBG sensors as well as the compressive strain in the midspan of the upper concrete surface. This last strain was captured with a strain gauge. The FBG strains shown in the figure were obtained once the temperature compensation had been carried out. The strain readings corresponding to FBG3 exhibited some abnormalities after the first load intervals; therefore, its evolution is only shown for the first tests. In the same way, the measurements captured from FBG2 were also abnormal once the steel reinforcement started to yield. The variation in the room temperature is suitably captured by FBG4. Higher temperatures clearly occurred during the summer months and the lowest temperatures correspond to the winter months. In the same way, four prominent peaks indicate when the heating of the specimen was carried out.

Figure 4 shows how the load increments along the time line are perfectly reflected by the strain increment experienced in all sensors. Similarly, the remanent compressive concrete strain increases gradually whenever the level of sustained load increases and the experimental program progresses. However, for the FBG2 sensor located at the midspan of the FRP, the remanent strain is lower after each unloading whenever the level of sustained load previously reached is higher. 

### 4.2. Impedance Signatures Measured from PZT

The impedance signatures were acquired only after each loading/temperature stage was completed and the specimen was unloaded. The PZT patches were interrogated at a frequency range of 10 to 100 kHz in steps of 12.5 Hz.

**Figure 5:**
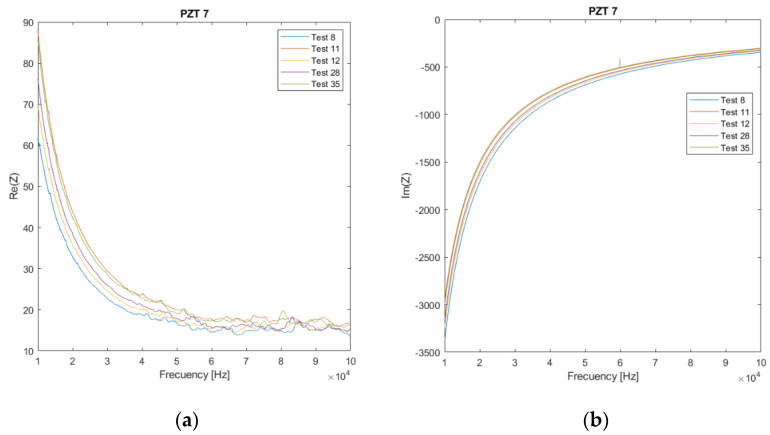
Impedance spectra for PZT7. (**a**) Real part; (**b**) Imaginary part.

Figure 5 shows the experimental impedance signatures of the specimen for PZT7, which was chosen as a sample. The real and imaginary parts are plotted. Considering that 35 EMI tests were performed, for simplicity, only some representative tests have been selected. In particular, tests 8, 11, 12, 28, and 35 are shown in Figure 5. Stages 8, 28, and 35 correspond to sustained load tests of 8, 13.7, and 19.6 kN, respectively, while stages 11 and 12 are associated with the heating of the beam and subsequent cooling. 

The real part of the impedance is clearly more sensitive to the changes in impedance signatures; therefore, it was used in the proposed technique. Impedance signatures shift in magnitude and frequency as the temperature changes and damage increases. Furthermore, changes associated with a temperature variation (heating in test 12) can be confused with damage (test 35), resulting in the detection of a false positive damage.

**Figure 6:**
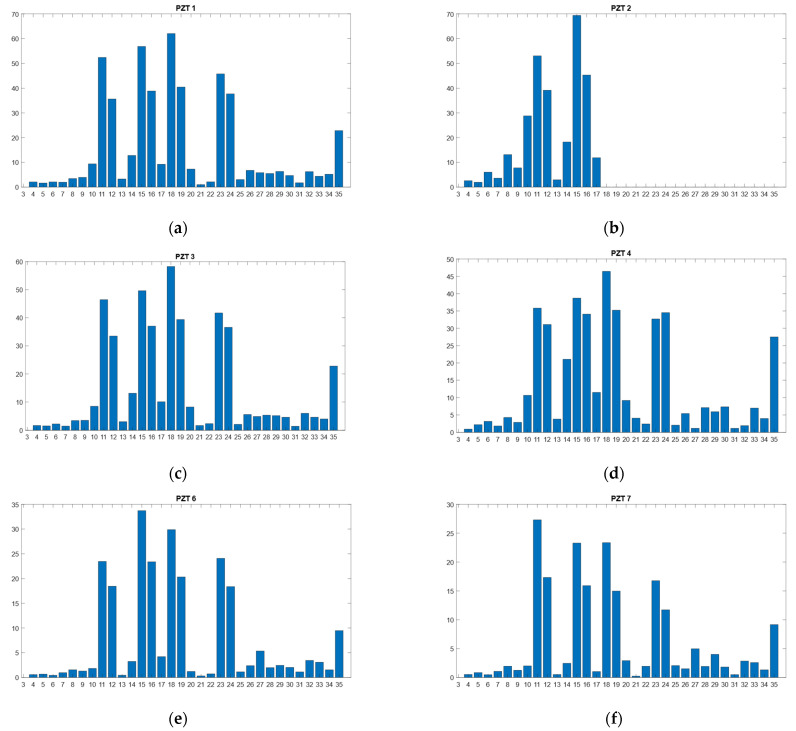

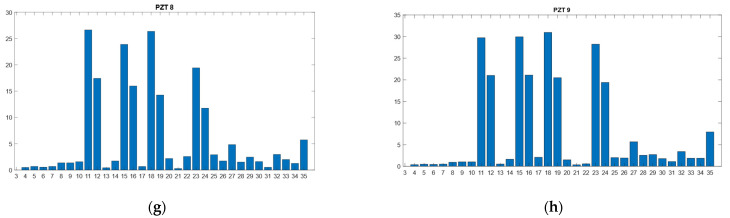
RMSD values for all PZT sensors. (**a**) Sensor PZT1, (**b**) Sensor PZT2, (**c**) Sensor PZT3, (**d**) Sensor PZT4, (**e**) Sensor PZT6, (**f**) Sensor PZT7, (**g**) Sensor PZT8, (**h**) Sensor PZT9.

The RMSD values calculated according to Equation (3) from the real part of the impedance signatures for all PZT sensors are shown in Figure 6. For the RMSD index, the larger the difference between the baseline reading and the subsequent reading, the greater the value of the index denoting greater changes in the impedance, which could be due to damage or thermal changes.

In accordance with Figure 3, Figure 6 clearly shows the high variation in the RMSD index due to the heating (tests 11, 15, 18, and 23) and the subsequent cooling until the environmental temperature is reached (tests 12, 16, 19 and 24). As RMSD is computed between consecutive tests, RMSD variations due to the temperature are less significant, except for those tests in which the temperature experiences a high and sudden variation. Another important aspect to note is that all sensors show a remarkable increase in test 35, which is a clear sign of deterioration in the specimen. Additionally, for the internal sensors, after the first sustained load test of 13.7 kN (test 27), a noticeable RMSD variation appears. For these sensors as well as for the external sensors, the changes in RMSD for test 27 and for the other three sustained load tests of 13.7 kN were very slight. If we take tests 28 and 29 as references, a variation of 3 °C occurred between both; however, in Figure 6, for all sensors, there is no clear evidence to suggest that this variation causes a remarkable change in the RMSD index. For some sensors (PZT1, PZT2, PZT3, and PZT6), the change is negligible, while for sensors PZT7 and PZT8, the change is probably due to the growth of internal damage near the midspan. The clustering analysis performed in the next section will help to confirm these conclusions. In any case, it is evident that the contribution of FBG is essential to effectively employ the EMI technique as the temperature variation can indicate false damage. 

## 5. Clustering Analysis

### 5.1. Hierarchical Clustering

**Figure 7:**
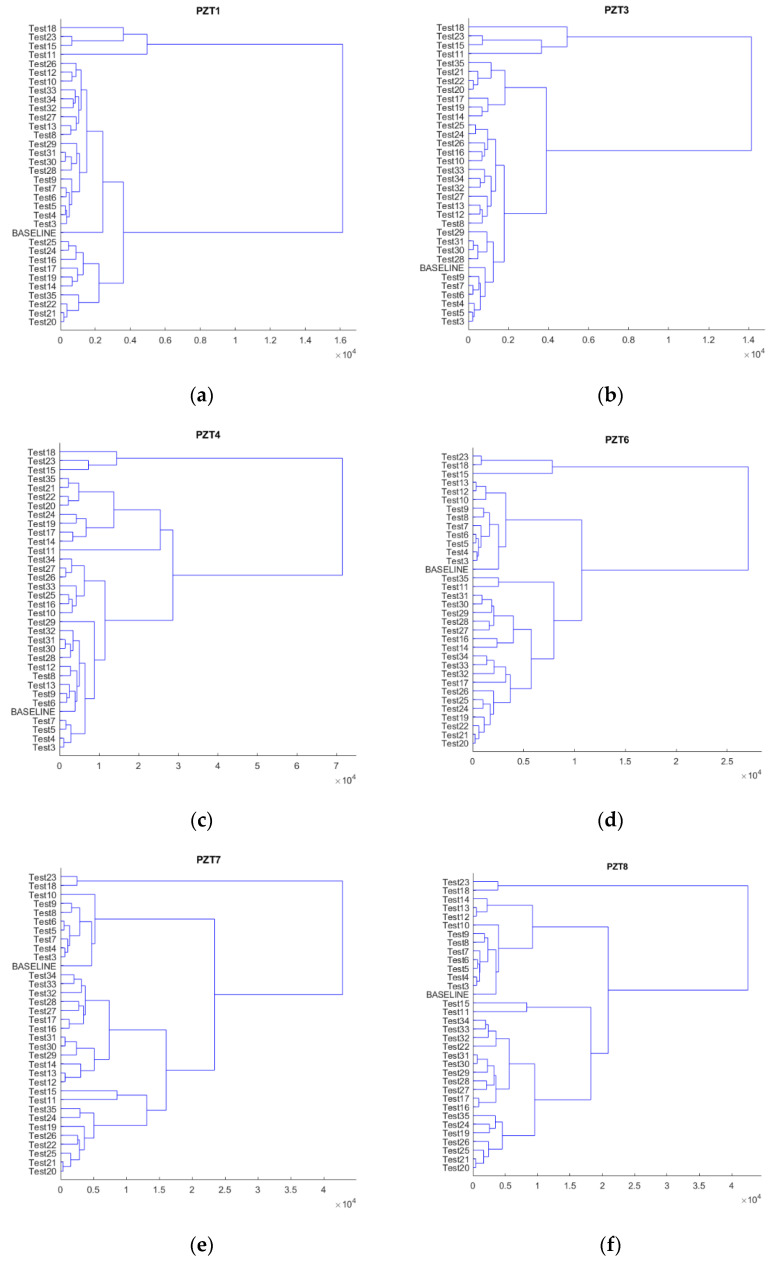

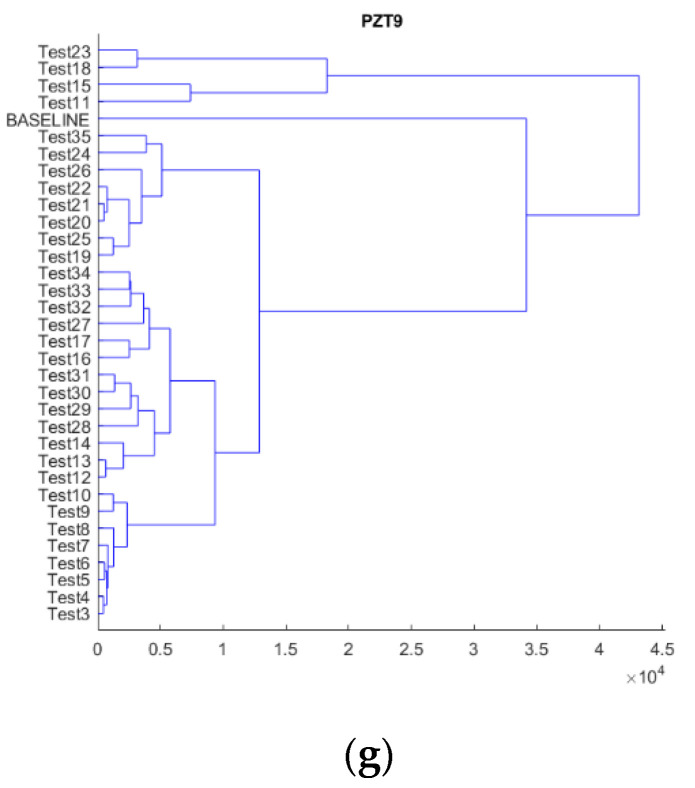
Hierarchical trees considering all tests. (**a**) PZT1, (**b**) PZT3, (**c**) PZT4, (**d**) PZT6, (**e**) PZT7, (**f**) PZT8, (**g**) PZT9.

The results derived from hierarchical clustering can be visualized as a dendrogram. Dendrograms show the hierarchical relationship between tests. Figure 7 shows the dendrograms constructed for each sensor and test by using the real part of the raw impedance data. Data from sensor PZT2 are not shown because of its malfunction in test 18. The length of the stems of the dendrogram represent the RMSD values at which the clusters are split up. By cutting the dendrogram at a particular length, we will have the number of clusters to be retained in the nested sequences of clustering that comprise the hierarchy. The height at which any two tests are joined together shows the order in which the clusters were joined according to its distance. In this way, for instance, the dendrogram in Figure 7a shows a significant difference between the cluster of test 11 and that of tests 15, 23, and 18 and between the cluster of 18 and that of tests 15 and 23.

The first conclusion from Figure 7 is that the heating tests (11, 15, 18, and 23) can be clearly identified, either because they are grouped in the same cluster or because the length of their stems stand out among the remaining tests. However, for the other tests, it is difficult to formulate any initial conclusions since the variations are of little significant. The number of tests is high and the variations among tests, except in some cases, are not very remarkable, which makes the process more difficult. 

Based on Figure 7, we might also state that stage 35 shows slightly different behavior compared to the other stages for the internal sensors. This phenomenon is not observed for the external sensors and might signify that the damage has become more severe in the internal areas of the beam around the FRP bar. However, to confirm this statement, additional analyses should be performed.

**Figure 8:**
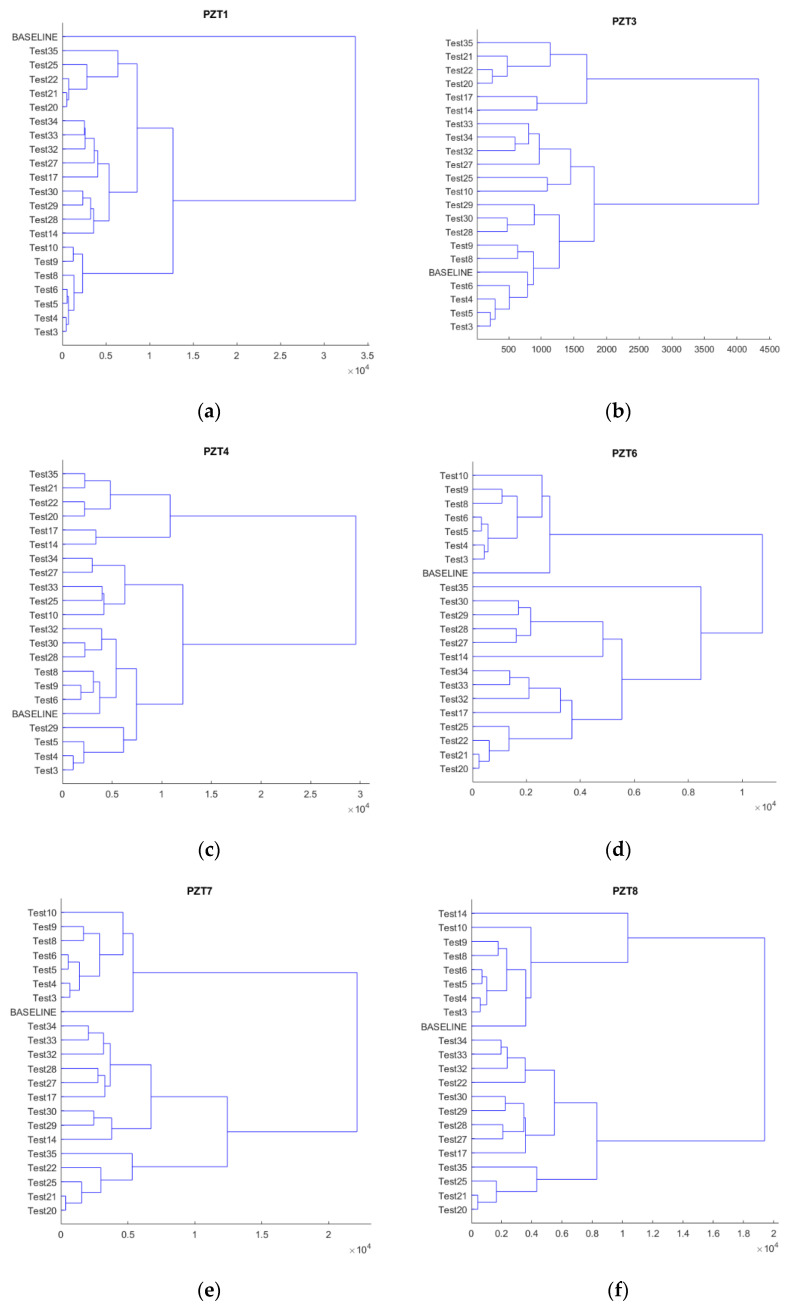

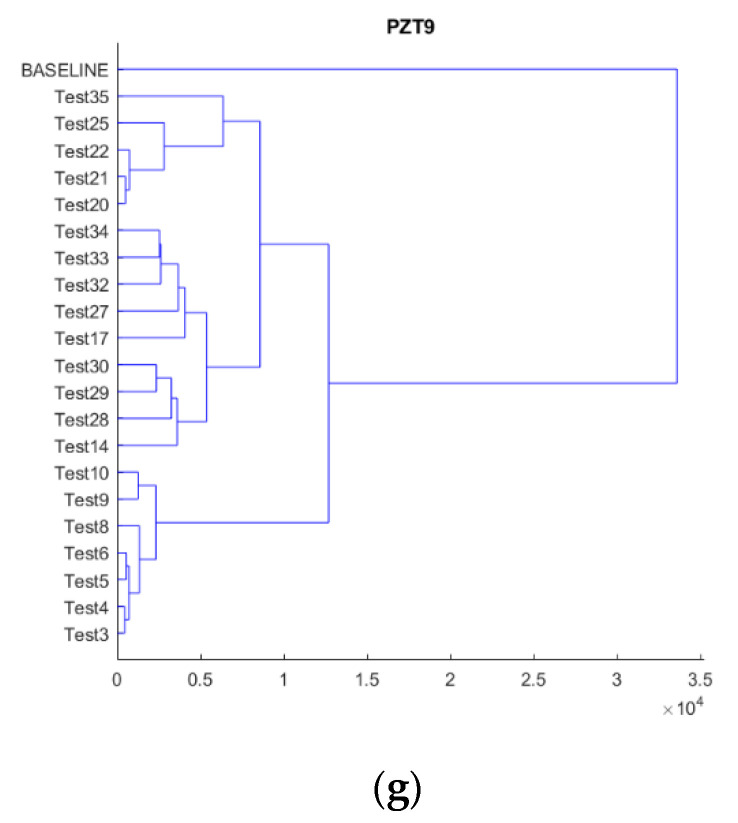
Hierarchical trees considering only tests subjected to load. (**a**) PZT1, (**b**) PZT3, (**c**) PZT4, (**d**) PZT6, (**e**) PZT7, (**f**) PZT8, (**g**) PZT9.

To get a deeper understanding of the results, an additional second level analysis was performed that considered only those tests under applied load, i.e., the heating and cooling tests were filtered in this second analysis. Figure 8 shows the results. It is clear that now, except for some particular tests, the different levels of the loads are gathered in the same group in agreement with the progressive degradation of the beam. The majority of these particular tests were those in which the beam was loaded after a heating–cooling sequence; this strong variation in the temperature affects the performance of the specimen. Furthermore, the internal sensors show a clear differentiation between state 35 and the rest of the states. This is especially true for sensor PZT6, which might be evidence of a sudden growth in the damage occurring in the last loading stage near this sensor. This phenomenon should be taken into account as a warning of the imminent failure of the beam.

### 5.2. K-Means Clustering

When k-means clustering is performed, the damage indicator shown in Equations (4) and (5) is computed for all observations. 

**Figure 9:**
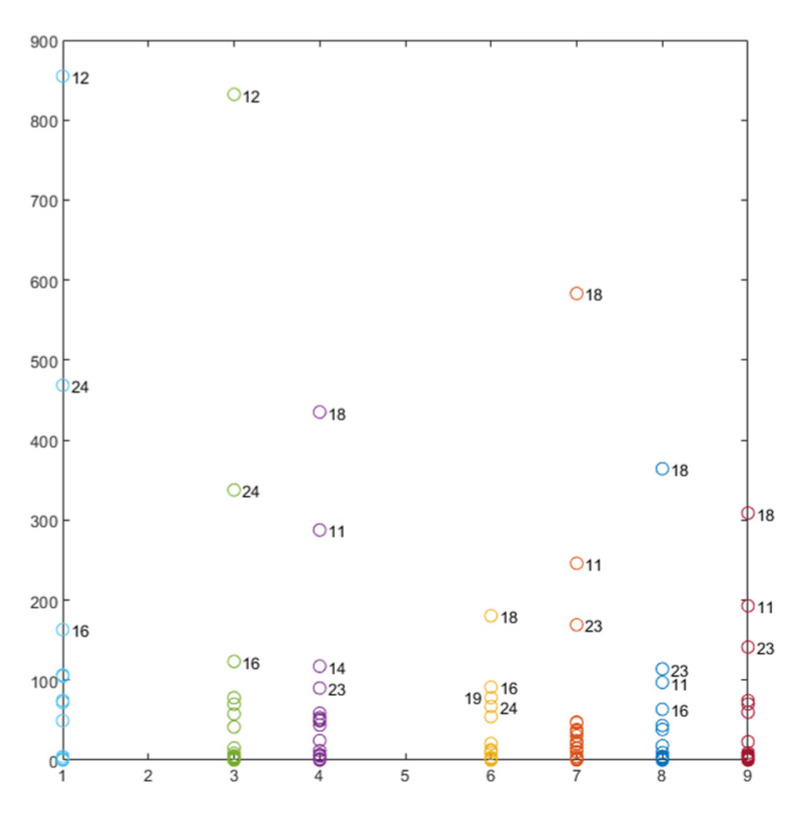
Damage index using the previous loading stage as the baseline.

Figure 9 shows the damage index of all the observations (all sensors and loading stages except baseline) computed between consecutive loading stages. This means that the baseline used for each test to compute D corresponds to the previous test. From the plot, it is clear that all anomalies are associated with the heating and cooling tests, i.e., those tests in which strong variations in temperature occurred. The predominance of these tests makes it difficult to detect any outliers due to the damage in the structure. Table 3 was created in order to analyze and interpret Figure 9 more deeply and to avoid confusion. In this table, the tests with the most severe damage, which were identified using Equation (5), are specified for each sensor. As expected, the main outliers are associated with the tests for which heating or subsequent cooling occur, which agrees with the peak values of the temperature FBG sensor in Figure 4. However, in Table 3, by filtering those heating and cooling tests, it is possible to identify the most critical tests from a mechanical damage point of view. Although it was not detected in Figure 6, test 34 can be identified as the most critical in the k-means analysis, which means that the accumulated damage might be an indication of possible future failure and shows the importance of using analyses that are complementary to the simple evaluation of the RMSD index. Temperature variations between this test and the previous one demonstrate that the effect of this variable is negligible and the detected anomaly is mainly due to the mechanical degradation of the specimen.

**Figure 10:**
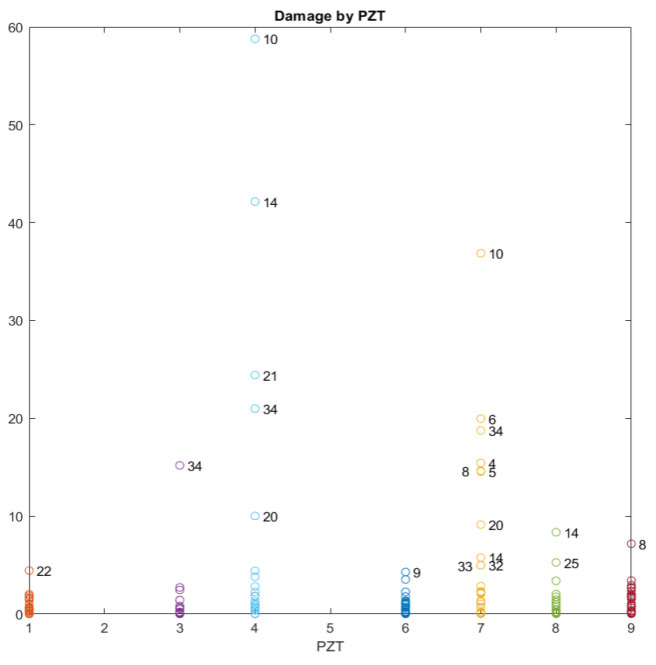
Damage index using the original state of the beam as the baseline.

To confirm the conclusions derived from Figure 9 and Table 3, damage indices were also computed taking the original healthy stage as the reference baseline for all tests. Furthermore, the k-means clustering used for the computation of the damage indices was obtained excluding those tests without any applied load. In this way, the heating and cooling tests were filtered in the study. As Figure 10 shows and in agreement with Figure 9, the results confirm that the state of the beam is more critical after test 34. Other tests where high damage indices were also obtained include those (14, 17, 20, and 25) in which the beam was reloaded after a heating–cooling cycle. We assume that this is due to the crack opening that occurs once the beam has been reloaded after having been unloaded for an extended period. 

## 6. Discussion and Conclusions

In this study, a clustering unsupervised machine learning system for SHM based on FBG and PZT measurements has been developed. Its performance was evaluated to identify minor damage by using the EMI method in the presence of sustained load and variable temperature. In a real scenario, the temperature variation can conceal real damage and it can also indicate false damage. The complementary nature of FBG and PZT smart sensors has been demonstrated to be key for developing a comprehensive SHM system applicable to a complex structural system in variable environmental conditions. The combination of multilevel hierarchical and k-means clustering techniques together with FBG measurements were used to provide a promising methodology for practical SHM applications.

Even though the method was applied in RC beams strengthened with the NSM-FRP technique, it is an effective and original contribution to any SHM system based on EMI. It allows us to address temperature variations and mechanical deterioration in a direct way, with low computational complexity, and without using any reference baseline signatures measured at different temperatures such as is done in the usual temperature variation compensation techniques. The standard temperature compensation techniques are applicable in a lab framework; however, their application on real structures is more limited because of the lack of reference patterns to implement the compensation. With the proposed method, the analysis is performed in a direct way using the support of FBG measurements and unsupervised machine learning techniques.

Despite the promising results, future work should assess the proposed approach in more depth when it is applied to more specimens considering other types of loads, such as fatigue loads and a narrower range of temperature variation. Additionally, the analysis of the performance of this method with other kinds of strengthening, such as EBR, should be carried out.

## Figures and Tables

**Table 1:** Properties of grade PIC151 PZT patches.

Property		PIC151
Density	ρ	7.80 g/cm^3^
Relative permittivity	ε_33_/ε_0_	2400
	ε_11_/ε_0_	1980
Dielectric loss factor	tanδ	0.02
Piezoelectric strain coefficient	d_31_	−210 × 10^−12^ C/N
	d_33_	500 × 10^−12^ C/N
Elastic compliance coefficient	$S_{11}^{E}$	15 × 10^−12^ m^2^/N
	$S_{33}^{E}$	19 × 10^−12^ m^2^/N

**Table 2:** Experimental protocol.

Test Number	dd/mm/yyyy	Sustained Load Level [kN]	Loading Test Duration [days]	Test Temperature [°C]	
0	08/01/2018	0	-	NA	NA
1	11/01/2018	8	2	17	Environmental
2	25/01/2018	8	7	19	Environmental
3	08/02/2018	8	14	17	Environmental
4	13/02/2018	8	4	17	Environmental
5	15/02/2018	8	1.5	17	Environmental
6	19/02/2018	8	3	18	Environmental
7	22/02/2018	0	3	18	Environmental
8	12/03/2018	8	14	19.5	Environmental
9	03/04/2018	8	21	19	Environmental
10	24/04/2018	8	21	22	Environmental
11	26/04/2018	0	1	42	Heated
12	30/04/2018	0	3	22	Environmental
13	03/05/2018	0	3	21	Environmental
14	27/05/2018	8	23	24	Environmental
15	31/05/2018	0	3	42	Heated
16	01/06/2018	0	2	24	Environmental
17	02/07/2018	8	30	27	Environmental
18	05/07/2018	0	3	47	Heated
19	09/07/2018	0	3	27	Environmental
20	25/07/2018	8	14	28	Environmental
21	26/07/2018	9.3	1	27.5	Environmental
22	04/09/2018	9.3	31	27	Environmental
23	05/09/2018	0	1	44	Heated
24	07/09/2018	0	3	27	Environmental
25	06/10/2018	9.3	21	25	Environmental
26	07/10/2018	13.7	1	22	Environmental
27	06/11/2018	13.7	28	20	Environmental
28	04/12/2018	13.7	29	20.5	Environmental
29	04/02/2019	13.7	60	17.4	Environmental
30	13/03/2019	13.7	41	19.5	Environmental
31	14/03/2019	17.7	1	19.5	Environmental
32	13/05/2019	17.7	60	22	Environmental
33	10/06/2019	17.7	30	24	Environmental
34	13/06/2019	19.6	2	24	Environmental
35	27/07/2019	19.6	42	29	Environmental

**Table 3:** Tests identified as having a higher damage index.

	Test Number from More to Less Severe									
PZT1	12	24	16	18	19	15	11	23	34	27
PZT3	12	24	16	19	18	11	15	23	34	14
PZT4	18	11	14	23	10	34	15	24	16	19
PZT6	18	16	24	23	19	11	12	15	27	28
PZT7	18	11	23	14	34	15	10	16	32	24
PZT8	18	23	11	16	24	19	15	12	34	32
PZT9	18	11	23	16	24	19	15	12	29	27

## Data Availability

The data presented in this study are available on request from the corresponding author. The data are not publicly available due to privacy reasons.
